# Hemangiopericytoma of the central nervous system: a case report

**DOI:** 10.11604/pamj.2023.44.58.36813

**Published:** 2023-01-31

**Authors:** Mohamed Moukhlissi, Sofia Elouaouch, Narjiss Aichouni, Soufiane Berhili, Loubna Mezouar

**Affiliations:** 1 Department of Radiotherapy, Hassan II Regional Oncology Center, Mohammed VI University Hospital, Mohammed First University, Oujda, Morocco,; 2 Department of Radiology, Mohammed VI University Hospital, Mohammed First University, Oujda, Morocco

**Keywords:** Hemangiopericytoma, cerebral, surgery, radiotherapy, case report

## Abstract

Hemangiopericytomas or solitary meningeal fibrous tumors are extremely rare mesenchymal tumors. They represent only 1.6% of all central nervous system tumors, occurring mainly in adults between 40 and 50 years of age with a slight male predominance. We report the observation of a 20-year-old man treated at the Oujda Regional Oncology Center for cerebral hemangiopericytoma, revealed by headaches resistant to usual analgesic treatments without other associated signs. Initial imaging a left temporo-parieto-occipital intraaxial tumor process all responsible for a sub-falcoial and temporal engagement whose appearance first evokes a high-grade glial tumor. The patient received a complete excision whose pathological examination with immunohistochemical study was in favor of a grade III hemangiopericytoma according to the WHO 2016 classification. Therapeutic management with adjuvant radiotherapy was supplemented with a volumetric modulated arc therapy (VMAT) technique at a total dose of 54Gy. We will discuss through this case, the clinical and therapeutic peculiarities by a review of the literature.

## Introduction

The term hemangiopericytoma (HPC) was first described in 1942 by Drs. Arthur Purdy Stout and Margaret Murray [1]. It is a rare, aggressive and highly vascularized mesenchymal tumor. As well, many features of HPC resemble meningiomas, which is why these were initially classified as angioblastic meningiomas. The 2016 WHO classification defines solitary fibrous tumor (SFTs)-HPC entities on a single spectrum with a single classification system. This is based on unique genetic events occurring in these pathologies, meaning the fusion of the NAB2 and STAT6 genes [2]. They are derived from fibro-histiocytic precursor cells: Zimmerman's pericytes [3]. The latter are immature spindle cells with contractile properties that attach to the capillary walls. Zimmerman cells are crucial for mechanically supporting capillaries, helping to change the light size during different physiological challenges. Compared to extracranial localization HPC, intracranial HPC is less common and remains a rare entity, accounting for 0.4% of all primary tumors of the central nervous system. Extracranial metastatic disease occurs more frequently in organs such as the bones, lungs and liver. These tumors are clinically similar to meningioma; however, they exhibit a faster growth time. They can cause symptoms due to the mass effect or local edema, with compression of the adjacent cerebral parenchyma and increased intracranial pressure. In this way, they usually experience symptoms such as headaches, focal neurological deficits, or comitial seizures [3,4].

## Patient and observation

**Patient information:** this is a young man aged 20 years, without a significant pathological history, with increasingly intense headaches and resistant to the usual analgesics evolving for a month before his first consultation without other associated signs, including no sensory-motor deficit.

**Clinical findings:** the patient was in good general condition, WHO=0 and the neurological examination was completely normal as well as the rest of the somatic examination.

**Figure 1:**
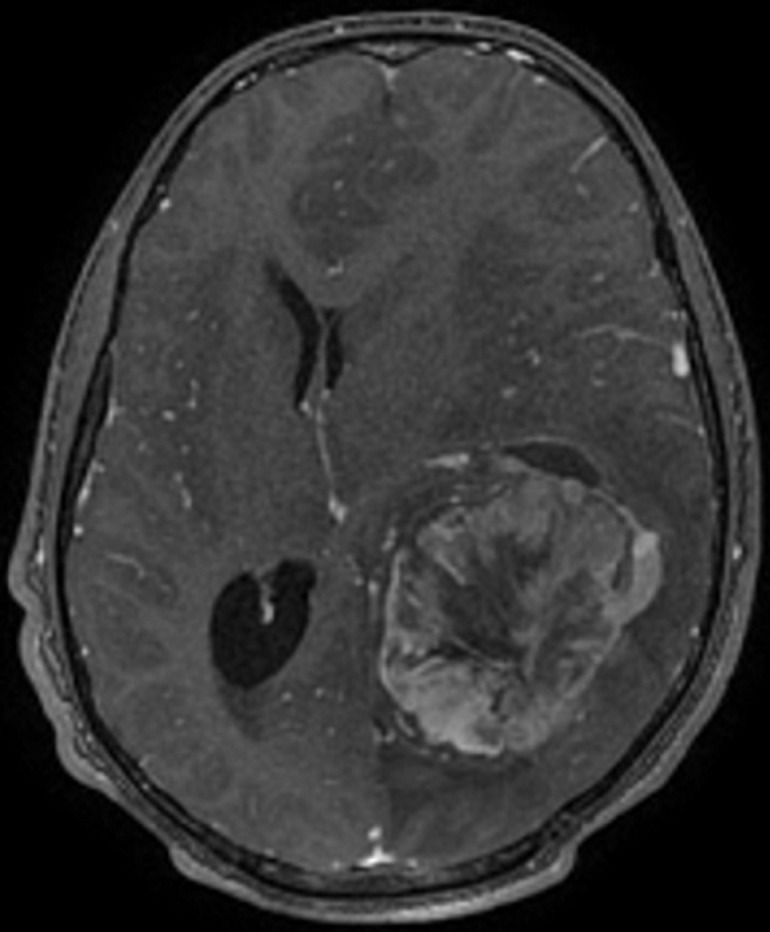
Magnetic resonance imaging (MRI) T1, objectified a left temporo-parieto-occipital intraaxial

**Figure 2:**
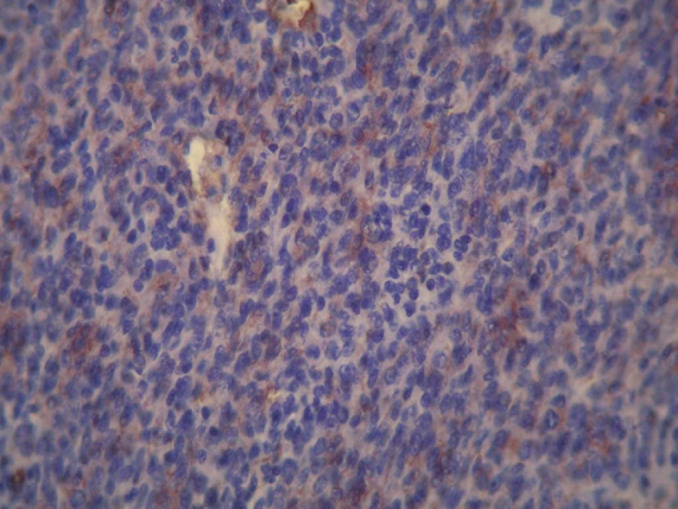
morphological and immunohistochimical appearance of a grade 3 malignant hemangiopericytoma of the 2016 WHO classification

**Diagnostic assessment:** initial imaging objectified a left temporo-parieto-occipital intraaxial tumor process responsible for a sub-falcoial and temporal engagement whose appearance first evokes a high-grade glial tumor (Figure 1). The patient received a complete excision whose pathological examination with immunohistochemical study was in favor of a grade III hemangiopericytoma according to the WHO 2016 classification (Figure 2).

**Therapeutic intervention:** after the tumor excision, a therapeutic management with adjuvant radiotherapy was supplemented with a VMAT technique at a total dose of 54Gy, with good tolerance.

**Figure 3:**
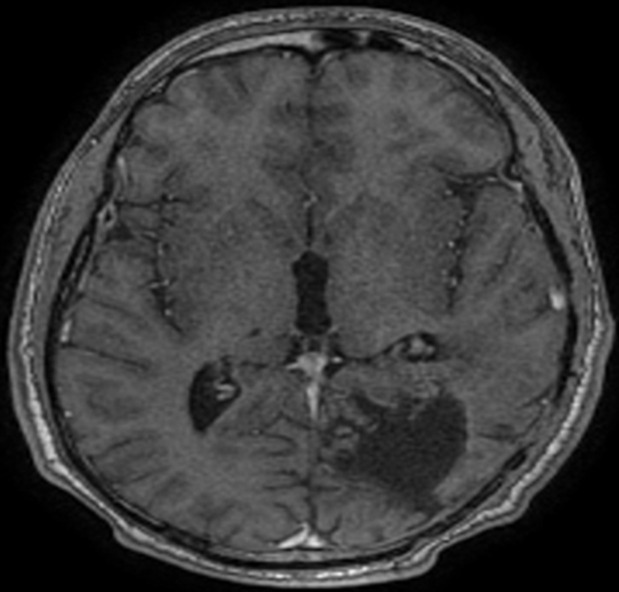
MRI T1, axial section showing the disappearance of the tumor

**Follow-up and outcomes:** control imaging 3 months after the end of radiotherapy did not show any peculiarity (Figure 3).

**Informed consent:** the patient is informed for this procedure of publishing a research paper.

## Discussion

Hemangiopericytomas or solitary meningeal fibrous tumors represent less than 1% of all CNS tumors, occurring mainly in adults between 40 and 50 years of age with a slight male predominance. It has supratentorial localization in the majority of cases (70%), followed by the posterior cerebral fossa (15%) and the spine (15%). The tumor has a similarity with meningioma on computed tomography and magnetic resonance imaging, but its clinical behavior is more aggressive with a high incidence of local recurrence, from 34% to 90%, and it is estimated that the follow-up of these patients should be carried out for at least 7.5 years. The percentage of long-range metastases is lower, from 12% to 55%, affecting in particular the bones, lungs and liver, in descending order [5]. Some studies suggest that the average time to local recurrence is about 5 years, while the average duration for distant metastases is about 8 years. This spread usually occurs through both lymphatic and hematogenous routes [5].

Enzinger and Smith histologically defined hemangiopericytoma as a tumor characterized by the presence of branched vessels, “staghorn”, often with perivascular hyalinization and abundant interstitial collagen, surrounded by small round to slightly spindle cells, and with no obvious clear microscopic characteristics of differentiation. It later became very clear that the morphological and immunohistochemical characteristics of hemangiopericytoma and solitary fibrous tumor are essentially identical, with classical hemangiopericytoma representing a more cellular and less highly collagenized variant. This concept is further validated by the discovery of identical NAB2-STAT6 protein fusions and nuclear expression of STAT6 in immunohistochemistry in tumors classified as hemangiopericytoma and solitary fibrous tumor in all anatomical sites, including meninges [6].

The World Health Organization has thus endorsed the term “solitary fibrous tumor” for this spectrum of lesions, “hemangiopericytoma” being considered an archaic label whose use is to be discouraged. According to the current WHO classification of 2016, HPCs are grouped with the solitary fibrous tumor, so that grade I most often corresponds to a highly collagenized fusiform cell lesion with relatively little cellularity previously diagnosed as a solitary fibrous tumor grade II generally corresponds to a tumor with more cellularity and less collagenized with plump cells and a vascularization “staghorn” or “deer antler” corresponded to the old hemangiopericytoma, and grade III most often corresponds to what was once called anaplastic hemangiopericytoma, with at least 5 mitoses for every 10 fields at high magnification.

Symptomatically, as described earlier, the symptoms are due to the mass effect exerted by the tumor, but also focal alterations, headaches or convulsions. It rarely exhibits intracranial bleeding, since only 12 cases have been described in the literature. On the microscopic level, the lesions show intratumor bleeding, because during the growth of the tumor, the consequences are: erosion of blood vessels, distortion, distension, occlusion and necrosis due to endothelial proliferation. In these special cases, emergency rescue surgery is required, as occurs in the case of subarachnoid hemorrhage [7].

It is essential to take into account tumor peculiarities when deciding on therapeutic choices. In case of aggressive tumor type, the treatment option should be less invasive and more palliative. Variables such as age, tumour size, number of mitoses (more than 5 per 10 fields at high magnification), retroperitoneal localization and incomplete excision of the primary lesion are associated with poor prognosis.

Currently, it could be part of the treatment of HPC: preoperative embolization, total or subtotal surgical resection of the tumor, adjuvant radiotherapy and stereotactic radiosurgery. However, chemotherapy is not effective in treating HPC, given its high aggressiveness, but may be considered in cases of peripheral metastases. The role of surgery, especially in the non-metastatic setting, is undisputed. As for the role of (adjuvant) radiotherapy, Ghia *et al*. [8] showed that in the subgroup of patients who underwent subtotal resection, the addition of postoperative radiotherapy had a dramatic effect on overall survival.

Also observed that, on multivariate analysis, postoperative radiotherapy was associated with significantly better survival, particularly for patients who underwent subtotal excision. This conclusion was confirmed by Kinslow *et al*. [9], stating that a combination of total tumour resection and radiotherapy was associated with significantly increased survival compared to total excision alone (HR = 0.537, p=0.039). In addition, patients treated with Gamma Knife equipment, are also available in the literature reporting favorable results as already mentioned, surgical excision and postoperative radiotherapy are widely recognized as effective treatments for HPC. Total excision is the main factor affecting the prognosis of HPC, which easily recurs in patients after surgery, with an average relapse time of 40 to 70 months. However, it has been reported that postoperative radiotherapy could reduce the rate of local relapse from 88% to 12.5% [10].

## Conclusion

Hemangiopericytoma has long been considered an aggressive and fatal tumor because of its resistance to treatment and its propensity for recurrence and metastasis. Due to its rarity, there is no standard for treatment. The combination of maximum cytoreduction surgery followed by adjuvant radiotherapy continues to be the most appropriate therapeutic choice for non-metastatic CHP. Localization in the central nervous system is still a rarity today, so randomized studies of a large number of cases are needed to arrive at a standard treatment plan.
